# Maternal Bisphenol A Exposure Promotes the Development of Experimental Asthma in Mouse Pups

**DOI:** 10.1289/ehp.0901259

**Published:** 2009-10-05

**Authors:** Terumi Midoro-Horiuti, Ruby Tiwari, Cheryl S. Watson, Randall M. Goldblum

**Affiliations:** 1 Department of Pediatrics, Child Health Research Center and; 2 Department of Biochemistry and Molecular Biology, University of Texas Medical Branch, Galveston, TX, USA

**Keywords:** airway hyperresponsiveness, asthma, bisphenol A, environmental estrogen, eosinophilia, experimental asthma, IgE, maternal exposure, perinatal sensitization

## Abstract

**Background:**

We recently reported that various environmental estrogens induce mast cell degranulation and enhance IgE-mediated release of allergic mediators *in vitro*.

**Objectives:**

We hypothesized that environmental estrogens would enhance allergic sensitization as well as bronchial inflammation and responsiveness. To test this hypothesis, we exposed fetal and neonatal mice to the common environmental estrogen bisphenol A (BPA) via maternal loading and assessed the pups’ response to allergic sensitization and bronchial challenge.

**Methods:**

Female BALB/c mice received 10 μg/mL BPA in their drinking water from 1 week before impregnation to the end of the study. Neonatal mice were given a single 5 μg intraperitoneal dose of ovalbumin (OVA) with aluminum hydroxide on postnatal day 4 and 3% OVA by nebulization for 10 min on days 13, 14, and 15. Forty-eight hours after the last nebulization, we assessed serum IgE antibodies to OVA by enzyme-linked immunosorbent assay (ELISA) and airway inflammation and hyperresponsiveness by enumerating eosinophils in bronchoalveolar lavage fluid, whole-body barometric plethysmography, and a forced oscillation technique.

**Results:**

Neonates from BPA-exposed mothers responded to this “suboptimal” sensitization with higher serum IgE anti-OVA concentrations compared with those from unexposed mothers (*p* < 0.05), and eosinophilic inflammation in their airways was significantly greater. Airway responsiveness of the OVA-sensitized neonates from BPA-treated mothers was enhanced compared with those from unexposed mothers (*p* < 0.05).

**Conclusions:**

Perinatal exposure to BPA enhances allergic sensitization and bronchial inflammation and responsiveness in a susceptible animal model of asthma.

The dramatic increase in the prevalence of childhood asthma in developed countries over the last two to three decades suggests that environmental changes related to industrialization may be involved in this epidemic (McCoy et al. 2005). One public health approach to understanding the asthma epidemic would be to identify environmental exposures that increased during the period when asthma prevalence began to increase in industrialized countries and to test their effects in experimental animals. Those compounds that enhance various features of asthma might be candidates for promoting the development or morbidity of asthma in humans. However, even the major reductions in some air pollutants accomplished in many areas have yet to have major impacts on the prevalence or even the morbidity of asthma. This experience suggests that the multifactorial nature of the disease makes it difficult to see the effects of incremental improvements in our environment when exposures to other types of toxicants persist or even increase.

We recently found that exposing cultured mast cells to estradiol (E_2_) strongly potentiates the synthesis and release of allergic mediators, acting through a membrane form of estrogen receptor α (ERα) (Zaitsu et al. 2007). We also examined the effects of environmental estrogens, alone and in combination with physiologic concentrations of E_2_, on the activation of a human mast cell line and primary cultures of murine mast cells (Narita et al. 2007). Like E_2_, low concentrations of environmental estrogens caused a rapid, partial degranulation of mast cells. Exposing HMC-1 cells (a human mast cell line) to a combination of suboptimal concentrations of E_2_ and an environmental estrogen had an additive effect on degranulation. Environmental estrogens also enhanced the release of β-hexosaminidase induced by allergen cross-linking of IgE (immunoglobulin E) on the surface of these cells. Bone-marrow–derived mast cells deficient in ERα expression had significantly reduced responses to some concentrations of environmental estrogens, suggesting that the degranulating activity of environmental estrogens on mast cells is mediated, at least in part, through ERα and that the dose response is nonmonotonic, as previously described for nongenomic responses to endogenous and environmental estrogens (Bulayeva and Watson 2004). Recent studies suggest that mast cells, in addition to their role as effects of allergic reactions, also contribute to the process of allergic sensitization (Nakano et al. 2009; Sayed and Brown 2007). We therefore examined whether environmental estrogens might enhance allergic sensitization and inflammation, thereby promoting the development of asthma and other allergic diseases.

To better understand the potential for environmental estrogens to promote the development of childhood asthma, in the present study we tested the hypothesis that prenatal and/or neonatal exposures to one of the most ubiquitous environmental estrogens, BPA, enhance the development of allergic asthma in a susceptible animal model. BPA is a substrate of polycarbonate plastics and has been produced in increasingly large quantities in industrialized countries since the 1950s. BPA is used to form plastic bottles, as a lining for food and beverage cans, and as a flame retardant and is a component of dental fillings. Interestingly, the increase of asthma prevalence among children started in the 1970s (Vollmer et al. 1998), 20 years (approximately one generation) after large-scale BPA production started in several industrialized regions of the world.

## Materials and Methods

### Animals

Female BALB/c mice were obtained from Harlan (Houston, TX) and were housed in pathogen-free conditions in the animal research facility of the University of Texas Medical Branch (UTMB; Galveston, TX) in accordance with the National Institutes of Health and UTMB institutional guidelines for animal care. Each mother and her litter were housed separately and fed a casein-based diet (Research Diet, New Brunswick, NJ) to eliminate estrogenic effects in the typical soy-based mouse diet from 1 week before BPA loading until the end of the study (Curran et al. 2004). We used animal cages made of polysulfone (Tecniplast, Buguggiate, Italy). We tested these cages for contamination with BPA by adding water to a cage and maintaining it for 1 week at room temperature. The concentration of BPA in the water was assayed with a highly sensitive gas chromatography–mass spectrometry (GC-MS) method (detection limit, 0.01 pg/mL). We found no detectable levels of BPA in the water. All experimentation was conducted under a protocol approved by the UTMB Institutional Review Board. The animals were treated humanely and with regard for alleviating suffering.

We compared the following study groups: *a*) BPA exposed and ovalbumin (OVA) sensitized/challenged (BPA-OVA); *b*) BPA exposed and not sensitized/challenged (BPA-PBS); *c*) not BPA exposed but OVA sensitized/challenged (N-OVA); and *d*) not BPA exposed and sham sensitized/challenged (N-PBS).

### BPA loading of female mice and pups

To simulate the relevant sources of BPA exposure in the human fetus and infant, we fed BPA (Sigma, St. Louis, MO) to the female mice before pregnancy. Female BALB/c mice were given 10 μg/mL BPA in 1% ethanol solution in their drinking water for 1 week before mating and throughout their pregnancy and lactation. We chose the concentration of BPA to feed the female mice based on a previous study (Kabuto et al. 2004) in which the body burdens of free BPA in the mothers and pups were similar to those described in human tissues and fluids. In that study, 5 or 10 μg/mL BPA in the mother’s drinking water resulted in 10^−8^ to 10^−7^ M concentrations in neonatal tissues, which is in the range reported from environmentally exposed humans (Table 1). Control female mice were given 1% ethanol solution in their drinking water from 1 week before mating until the end of the study. All of the outcome measures were performed only on pups.

### Allergic sensitization and exposure

To detect effects of BPA on allergic sensitization, we gave suboptimal doses of OVA to the pups, as described previously (Fedulov et al. 2008; Hamada et al. 2007). A single intraperitoneal (ip) injection of 5 μg OVA with 1 mg alum (Sigma) as an adjuvant was given on postnatal day 4 (PND4). These mice were subsequently exposed to aerosolized 3% OVA solution in phosphate-buffered saline (PBS) for 10 min on PNDs 13, 14, and 15, using a jet nebulizer (Pari II; Pari Industries, Richmond, VA). Markers of the development of asthma were assessed on PND17.

### Pulmonary function testing

To assess airway hyperresponsiveness (AHR), we used whole-body barometric plethysmography (Buxco Electronics, Sharon, CT) 48 hr after the OVA challenge on PND17 (Leme et al. 2006) as previously described (Castro et al. 2006). Measurements of airway responses were performed on individual, unrestrained, nonanesthetized mice in a four-chamber plethysmograph. AHR was expressed as an enhanced pause (Penh), a dimensionless parameter used to measure pulmonary resistance, calculated from changes in the pattern of chamber pressure induced by methacholine challenge. After a brief acclimatization in the chamber, the mice received an initial baseline challenge of saline, followed by increasing doses of nebulized methacholine (1, 10, 25, and 50 mg/mL). Recordings were taken for 3 min after each nebulization. The respiratory rate in breaths per minute was extrapolated from readings of every 10 breaths. The box pressure waveforms generated from the respiratory cycle were used to calculate peak expiratory pressure (PEP), peak inspiratory pressure (PIP), and the time of expiration. Penh was then calculated using the formula Penh = pause × PEP/PIP. Penh values were averaged and reported as a percentage of baseline nebulized saline inhalation.

Some of the pups from each treatment group were analyzed using the forced oscillation technique (flexiVent; SCIREQ, Montreal, Quebec, Canada) to validate the results of the whole-body barometric plethysmography, as described previously by Shore et al. (2003). Briefly, we treated these animals similarly to those described above, except we gave them aerosolized 3% OVA on PNDs 18–20. On PND22 (48 hr after the last aerosol challenge), animals were anesthetized with xylazine (7 mg/kg) and pentobarbital sodium (50 mg/kg). After performing a tracheostomy, we used a tubing adaptor (19 gauge; Becton-Dickinson, Franklin Lakes, NJ) to cannulate the trachea and then ventilated the mice at 150 Hz with a tidal volume of 0.3 mL. Pressure at the airway opening was measured by the flexiVent system. We applied a positive end-expiratory pressure of 3 cm H_2_O. We used a 2.5-Hz sinusoidal forcing function to measure dynamic pulmonary resistance (RL) by the forced oscillation technique. We obtained dose–response curves to nebulized methacholine (0.1, 1, 10, 30 and 50 mg/mL); the five highest values of RL obtained after each dose were averaged to obtain the final values for each dose.

### Quantifying the inflammatory cells in bronchoalveolar lavage (BAL) fluid

On PND17, mice were sacrificed, and total leukocytes and eosinophils in BAL fluid were counted. Immediately after sacrifice, cells in the lungs were recovered by flushing the isolated trachea with 0.5 mL PBS twice. Total leukocytes were counted using a hemocytometer. Eosinophil counts were calculated from differential cell counts in 150 μL fluid deposited onto glass slides using a Cytospin 3 centrifuge (400 × *g* for 4 min; Shandon Lipshaw, Pittsburgh, PA) and stained with hematoxylin and eosin (Anderson and Poulsen 2003). The results were expressed as the absolute number of total cells and eosinophils.

### Quantification of OVA-specific antibodies in mouse sera

To evaluate the effects of the environmental estrogen BPA on *in vivo* sensitization, we measured allergen-specific IgE and IgG1 in the sera of the mouse pups at the time of sacrifice. Individual measurements of IgE and IgG1 were performed by ELISA (enzyme-linked immunosorbent assay). We used OVA-specific monoclonal IgE and IgG1 antibodies (Gene Tex, Inc., San Antonio, TX) as standards, and biotinylated anti-mouse IgE (R&D Systems, Minneapolis, MN) and horseradish peroxidase anti-mouse IgG (H&L; Zymed, San Francisco, CA) for detection. The detection limit for both IgE anti-OVA and IgG anti-OVA was 5 ng/mL. Values below the detection limit are shown on the baseline.

### Statistical analysis

Results are expressed as the mean ± SE. Statistical analysis was performed using one-way analysis of variance. Where differences between groups were present, they were further analyzed by the Student’s *t*-test. A *p*-value of < 0.05 was defined as statistically significant.

## Results

### The effect of BPA exposure on pregnancy

We found no significant differences in the BPA-exposed and unexposed mothers in time to impregnation, litter size, birth weights, or sex ratio of the pups (data not shown).

### Effect of BPA on allergen-induced AHR

**Figure 1:**
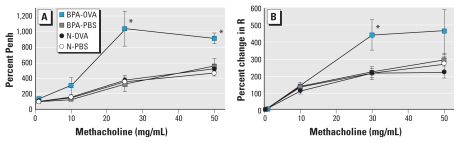
BPA effects on AHR after methacholine challenge. The lung function of the pups was analyzed by (*A*) whole-body barometric plethysmograph on PND17 (challenged with 0, 1,10, 25, or 50 mg/mL methacholine) or (*B*) forced oscillation analysis on PND22 (challenged with 0, 0.1, 1, 10, 30, or 50 mg/mL methacholine). R, lung resistance. Data are mean ± SE (*n* = 7–11). Analysis was performed on pups from 4–6 mothers and divided into OVA and sham immunization groups. *p < 0.05 compared with all other groups after 25 mg/mL and 50 mg/mL methacholine by whole-body barometric plethysmograph and after 30 mg/mL by the forced oscillation analysis.

Figure 1 shows the effect of BPA exposure on the development of AHR as assessed by whole-body barometric plethysmograph and the forced oscillation technique. Airway responsiveness to methacholine was significantly increased in BPA-OVA pups compared with all other groups using both methods of analysis. These differences were present in response to both 25 and 50 mg/mL methacholine when measured by whole-body barometric plethysmograph and 30 mg/mL by the forced oscillation technique. The patterns of Penh and lung resistance responses to methacholine were quite similar. This result is consistent with a previous report by Adler et al. (2004) in BALB/c mice that showed a significant correlation between Penh and lung resistance.

### Effect of BPA on pulmonary inflammation

**Figure 2:**
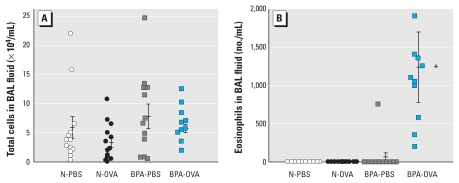
BPA effects on OVA hypersensitivity. Total cell number (*A*) and eosinophil counts (*B*) in BAL fluid 13 days after ip injection with OVA or PBS. The result for each pup is shown as an individual point (*n* = 12–16 pups from 6–7 mothers per group), and the bars indicate the mean ± 1 SE for groups. *p < 0.05 compared with all other groups.

**Figure 3:**
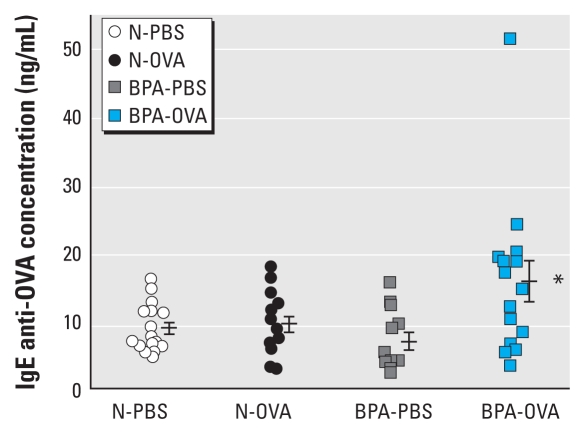
BPA effects on allergen-specific IgE production. IgE anti-OVA concentrations in sera were measured by ELISA. The result for each pup is shown as an individual point (*n* = 12–16 pups from 6–7 mothers per group), and the bars indicate mean ± 1 SE for groups. The detection limit was 0.05 ng/mL. *p < 0.05 compared with all other groups.

To determine whether BPA alters allergen-induced pulmonary inflammation, we quantified total and differential cell counts in BAL fluid. We observed a significant increase in eosinophils in BAL fluid from BPA-OVA pups (Figure 2B) compared with all other groups. We found no significant difference in the total cell number between the groups (Figure 2A). We derived the data for each group from 6–7 mothers and 12–16 pups. Pups from each litter were distributed in both the OVA-sensitized and nonsensitized groups, so the unit of analysis was the individual pups. Statistical analysis was performed with and without the inclusion of the BPA-OVA outlier shown in Figure 3. Both analyses showed *p* < 0.05 for eosinophil concentration in BAL fluid from BPA-OVA pups compared with all other groups.

### Effect of BPA on allergen-specific antibody production

The concentration of IgE anti-OVA antibodies in the sera from BPA-OVA pups was significantly higher than that for the other three groups (*p* < 0.05; Figure 3). IgG1 anti-OVA concentrations in sera from these four groups of pups did not differ. Statistical analysis performed with and without the outlier (BPA-OVA pup) showed statistical significance (*p* < 0.05) for IgE anti-OVA in the sera from BPA-OVA pups compared with all other groups, but no significance for IgG anti-OVA concentrations. The outlier in Figure 3 was the same BPA-OVA pup with the high eosinophil number in the BAL fluid (Figure 2B).

## Discussion

To begin testing the hypothesis that exposure of genetically susceptible animals to an environmental estrogen during critical periods of immune development promotes allergic sensitization and enhances subsequent inflammatory reactions, we chose to analyze the responses of neonatal mice to BPA exposure during the perinatal period. We found that AHR, eosinophilic inflammation, and allergen-specific IgE were all significantly increased in the allergen-sensitized/challenged, BPA-exposed pups compared with those that were not exposed to BPA or not sensitized to OVA. These findings are quite consistent with our hypothesis. The BALB/c strain of mice is considered to be susceptible to allergic sensitization and has been used extensively as a model of allergic asthma after sensitization with OVA. However, most studies of OVA-induced asthma in BALB/c mice have started the sensitization process later in life and have used multiple and larger ip doses of OVA to prime the allergic response and larger inhaled doses to complete the sensitization and to induce airway inflammation and hyperreactivity. We chose an intentionally “suboptimum” sensitization protocol that was designed and used extensively by Kobzik and colleagues (Fedulov et al. 2008; Hamada et al. 2007) to examine maternal influences on development of the asthma phenotype in neonatal BALB/c mice. This approach allowed us to demonstrate in our model that early life exposure to BPA enhanced the development of asthma under conditions that otherwise would not induce this phenotype. However, the present study does not exclude the possibility that exposure to BPA *in utero* only, during early infancy only, or even in adulthood may have similar effects on the development or manifestations of asthma.

Our finding that BPA exposure enhanced allergic sensitization (serum IgE anti-OVA antibodies), eosinophilic airway inflammation, and bronchial hyperreactivity does not allow us to distinguish primary from secondary or tertiary effects of BPA on the development of these components of the asthma phenotype. However, because exposure to both BPA and OVA was required to induce AHR in our model, it seems very likely that allergic sensitization is required for BPA to promote asthma development, although sensitization alone may not be sufficient to induce the asthma phenotype. This proposition is in keeping with the study by Ohshima et al. (2007) that found that BPA exposure in mice also promotes the manifestation of food allergy in orally sensitized mice. Our finding that our BPA exposure/OVA sensitization protocol did not cause an increase in mouse IgG1 antibodies to OVA [another T-helper cell (T_H_2)-driven isotype] may be explained by the recent finding that IgG1 is a switch intermediate between IgM and IgE expression (Sudowe et al. 1997). Because BPA has been shown to induce production of a large amount of interleukin-4 (IL-4) (Johnson et al. 1996; King et al. 1998; Siegrist 2001), this may drive the IgG1-expressing B cells on to IgE expression. However, this finding may also be a manifestation of the immaturity of the B-cell system at this age (Johnson et al. 1996; King et al. 1998; Siegrist 2001).

The key cell types and mechanisms by which BPA promotes allergic asthma in mouse pups remain to be elucidated. However, a number of sex steroid effects on immune system functions have been described (Watson and Gametchu 2001), although relatively few have been explored mechanistically. Estrogens (10^−7^ M E_2_, nonylphenol, and octylphenol) promote a T_H_2 response associated with increased IL-4 and decreased interferon-γ (IFN-γ) production from CD4^+^CD8^+^ thymocytes and naive CD4^+^ T cells isolated from C57BL/6 mice (Iwata et al. 2004). Lambert et al. (2005), using cells from an ERα knockout mouse on a C57BL/6 background, found that 10^−9^ M E_2_ acts through ERα to increase IL-4 and GATA-3 expression and essential T_H_2 cytokine and transcription factors in CD4^+^ cells and to reduce IFN-γ production from macrophages. E_2_ (10^−8^ to 10^−6^ M) also has effects on major histocompatibility complex class II expression on spleen dendritic cells isolated from BALB/c mice (Yang et al. 2006). High concentrations of BPA (10^−5^ to 10^−4^ M) and nonylphenol (10^−7^ to 10^−6^ M) have been shown to induce IL-4 and IgE production from CD4^+^ T cells (Lee et al. 2003).

Environmental toxins are encountered to varying extents in both industrialized and developing nations and rural and urban environments. Some of these compounds are by-products of industrial plants or agriculture, such as pesticides. They often contaminate water supplies, exposing fish and other animals, including humans. Although the World Health Organization strongly supports breast-feeding, breast-milk monitoring studies suggest that environmental chemicals that may affect children’s health are transmitted through breast-feeding (Solomon and Weiss 2002; Wang et al. 2004). This is especially true for environmental lipid-soluble pollutants such as polyhalogenated compounds, because these chemicals tend to degrade slowly in the environment, bioaccumulate and bioconcentrate in the food chain, and have long half-lives in humans. Because the fat content of breast milk is relatively high, the concentration of some of these pollutants is 100 times higher in maternal milk than in plasma (Dewailly et al. 1993). As the final consumers in the food chain, human infants may consume the highest concentrations of lipid-soluble environmental pollutants, which might enhance their risk of developing asthma or other allergic diseases due to the actions of environmental estrogens.

Newborns generally have a T_H_2-skewed pattern of immunity, partially due to low production of IL-12 and the propensity of T_H_1 cells to undergo apoptosis after antigen exposure (Allam et al. 2005; Prescott et al. 1998). Subsequently, “immune-maturing” infections are thought to promote a shift toward T_H_1 responses in most children, whereas some remain prone to develop T_H_2 responses (Prescott et al. 1999) and their disease consequences. In keeping with this progression, most cases of asthma develop in early childhood. Thus, environmental exposures *in utero* and during first months of life are likely to be very important (Holt and Jones 2000). Further, Gern et al. (1999) found that asthma, at any age, is likely to originate in childhood or earlier. Therefore, immunologic events in children and experimental models of airway injury and repair in early life may provide important clues to the inception and pathogenesis of asthma.

We observed that our adult mice drank about 5 mL of 10-μg/mL BPA in their water and therefore estimate that they consumed about 2 mg/kg/day of BPA. Lee et al. (2003) reported that injection of keyhole limpet hemocyanin (100 μg) into the footpad followed by BPA (25 mg/kg/day) or nonylphenol (5 mg/kg/day) by ip injection induced production of both IL-4 and IgE in adult mice. It is possible that the mice in their study had higher tissue levels of BPA than did ours, because we found that the range of environmental estrogens that induced mast cell degranulation *in vitro* was somewhat broad, 10^−11^ to 10^−9^ M (Narita et al. 2007), suggesting that a broad range of BPA may also promote allergic sensitization *in vivo*.

The results described here indicate that we must give due consideration to the possible impact of environmental estrogens on normal immune development and on the development and morbidity of immunologic diseases, such as asthma. More extensive studies are required to analyze the cellular and molecular mechanisms that underlie these effects during specific developmental windows and thus identify approaches to prevent exposures or remediate effects of the xenoestrogens. Understanding the implications of our study for human asthma will require epidemiologic studies that examine the effect of BPA burden of mothers and their children on the risk of developing childhood and adult asthma in large populations.

## Figures and Tables

**Table 1:** Reported free BPA concentrations in human samples.

Sample	Location	Method	Free BPA level (ng/mL)	Reference
Plasma, pregnant	USA	HPLC	0.5–22.3	Padmanabhan et al. 2008
Plasma	Germany	LC-MS	< 0.5	Volkel et al. 2005
Plasma, maternal	Germany	GC-MS	0.3–18.9	Schonfelder et al. 2002
Plasma, cord	Germany	GC-MS	0.2–9.2	Schonfelder et al. 2002
Serum, maternal	Japan	HPLC	0.46 ± 0.20	Kuroda et al. 2003
Serum, cord	Japan	HPLC	0.62 ± 0.13	Kuroda et al. 2003
Serum	Belgium	GC-MS	0.98 ± 1.09	Dirtu et al. 2008
Urine	USA	HPLC	< 0.3–0.6	Ye et al. 2005
Urine, men	Korea	HPLC	0.28–2.36	Kim et al. 2003
Urine, women	Korea	HPLC	0.068–1.65	Kim et al. 2003
Urine	Japan	GC-MS	0.01–0.27	Tsukioka et al. 2003
Urine	Germany	LC-MS	< 1.14	Volkel et al. 2005
Breast milk	Japan	HPLC	0.61 ± 0.20	Sun et al. 2004
Adipose tissue, women	Spain	GC-MS	5.83 ± 3.48	Fernandez et al. 2007
Placenta	Germany	GC-MS	1.0–104.9	Schonfelder et al. 2002

Abbreviations: HPLC, high-performance liquid chromatography; LC-MS, liquid chromatography–mass spectrometry.
